# Evaluating BDNF Levels in Plasma and its Relationship to Established Biomarkers of Alzheimer’s Disease

**DOI:** 10.1002/alz.090866

**Published:** 2025-01-09

**Authors:** Paul J Kueck, Robyn A Honea, Riley E Kemna, Jeffrey M Burns, Jill K Morris

**Affiliations:** ^1^ University of Kansas Alzheimer's Disease Research Center, Fairway, KS USA

## Abstract

**Background:**

Plasma biomarkers show a promising future to improving the quality of diagnosing Alzheimer's Disease (AD). However, blood processing procedures should be considered when measuring plasma biomarkers. Here we investigate brain‐derived neurotrophic factor (BDNF) in platelet‐rich and platelet‐poor plasma. Furthermore, we examine the relationship between BDNF and pTau181, a known biomarker of AD, in comparison to healthy older adults (CH).

**Method:**

98 participants (21 AD) completed a clinical and cognitive examination, a fasting blood draw and MRI scan. Participants with cognitive impairment other than dementia were excluded. Cognitive scores were standardized to yield a global z‐score. EDTA platelet‐rich plasma (PRP) was centrifuged 1500xg for 10min, while platelet‐poor plasma (PPP) was generated by immediately centrifuging a second time at 1700xg for 15min. Biomarker analyses were performed on Simoa HD‐X (Quanterix). T1‐weighted images were collected on a Siemens 3.0T scanner. Gray matter volume (GMV) was calculated using CAT12 toolbox and normalized to total intracranial volume. Log transform was used to normalize biomarker data. Diagnostic differences were assessed using ANOVA controlling for age and sex, and relationships between continuous variables were analyzed via linear regression.

**Result:**

There were no differences in age and sex between groups. As expected, AD global cognitive z‐scores (p<0.001) and GMV (p<0.001) were lower compared to CH. BDNF measured from PRP was significantly greater than PPP (p<0.001). Group differences were not observed in PRP BDNF (p=0.765), and AD were trending to have lower PPP BDNF (p=0.075) compared to CH. AD were observed to have elevated pTau181 (p=0.015) compared to CH. pTau181 showed a negative association with GMV across both groups (β=‐0.252, p=0.012). PPP BDNF showed a positive association to pTau181 in the AD (β=0.645, p=0.008) but not in the CH.

**Conclusion:**

Platelets can release BDNF and thus it’s important to remove during processing to better reflect circulating BDNF levels. BDNF protects against tau‐related neurodegeneration in animal models. Here we see cross‐sectionally a relationship between BDNF and pTau181. The positive association may reflect a compensatory mechanism, given BDNF’s known role in neuroplasticity. Further research is needed to better understand the possible mechanism involved and the long‐term relationship between these biomarkers.

